# Pseudomyxoma peritonei and an incidental low-grade appendiceal mucinous neoplasm and neuroendocrine appendiceal collision tumour: a case report

**DOI:** 10.1093/jscr/rjad281

**Published:** 2023-05-25

**Authors:** Ankit Gupta, Lavesh Mirpuri, Faizan Malik, Hussain Hassan, Nasira Amtul

**Affiliations:** School of Medicine, University of Leeds, Worsley Building, Woodhouse, Leeds LS2 9JT, UK; School of Medicine, University of Leeds, Worsley Building, Woodhouse, Leeds LS2 9JT, UK; School of Medicine, University of Leeds, Worsley Building, Woodhouse, Leeds LS2 9JT, UK; School of Medicine, University of Leeds, Worsley Building, Woodhouse, Leeds LS2 9JT, UK; Leeds Institute of Emergency General Surgery, St James's University Hospital, Beckett Street, Leeds LS9 7TF, UK

**Keywords:** Pseudomyxoma peritonei, general surgery, appendiceal cancer, neuroendocrine tumour

## Abstract

Appendiceal collision tumours are extremely rare, with most reported cases describing tumours consisting of a mucinous component and a neuroendocrine component. Low-grade appendiceal mucinous neoplasms, in some cases, have a tendency to rupture and disseminate their mucin-producing cells throughout the abdominal cavity, leading to a clinical syndrome known as pseudomyxoma peritonei (PMP). We present the case of a 64-year-old male who initially presented with acute appendicitis and was subsequently found to have PMP and appendiceal malignancy. After several years of scans, surgical intervention and histological analysis, it became apparent that the appendiceal malignancy was comprised of distinct cell types. The patient underwent two rounds of cytoreductive surgery with hyperthermic intraperitoneal chemotherapy, which resulted in a 2-year disease-free period. Unfortunately, the PMP recurred, having morphological changes consistent with a more aggressive disease process.

## INTRODUCTION

Appendiceal collision tumours are an extremely rare clinical entity, having only been described in a handful of published reports. Pseudomyxoma peritonei (PMP) is also a rare pathology, with an estimated incidence of 1 per million per year [1]. As a rare disease, early and accurate diagnosis is often difficult, leading to patients presenting quite late with features of clinically advanced disease. There is a lack of consensus surrounding the definitive aetiology of PMP; however, the most accepted notion is that the rupture of a low-grade appendiceal mucinous neoplasm (LAMN) causes dissemination of mucin throughout the peritoneal surface [2].

We report an extremely rare case of PMP, coexisting alongside an incidental LAMN and neuroendocrine appendiceal collision tumour.

## CASE REPORT

A 64-year-old male presented to the Emergency Department with a short history of abdominal pain. On examination he was tender and exhibited guarding in the right iliac fossa. Clinically, he was diagnosed with appendicitis and underwent a computed tomography (CT) scan. The CT scan demonstrated findings in keeping with acute appendicitis, however, also noted extensive caecal thickening with multiple reactive lymph nodes in the adjacent mesentery. The reporting radiologist also commented on an ill-defined 2.3 cm subphrenic opacity, as likely to be of no significance, but the possibility of a peritoneal nodule should not be ruled out. This episode was managed conservatively.

In view of the initial CT findings, a colonoscopy was performed which ultimately revealed no abnormalities in either the mouth of the appendix or the caecum. Multiple biopsies were taken that showed no evidence of dysplasia or malignancy. A repeat CT at 3 months showed considerable improvement in the appearances of the appendix, caecum and adjacent mesentery. Some residual thickening of the caecal pole and appendix remained, but there was no evidence of intraperitoneal metastatic disease or other cause for concern.

**Figure 1:**
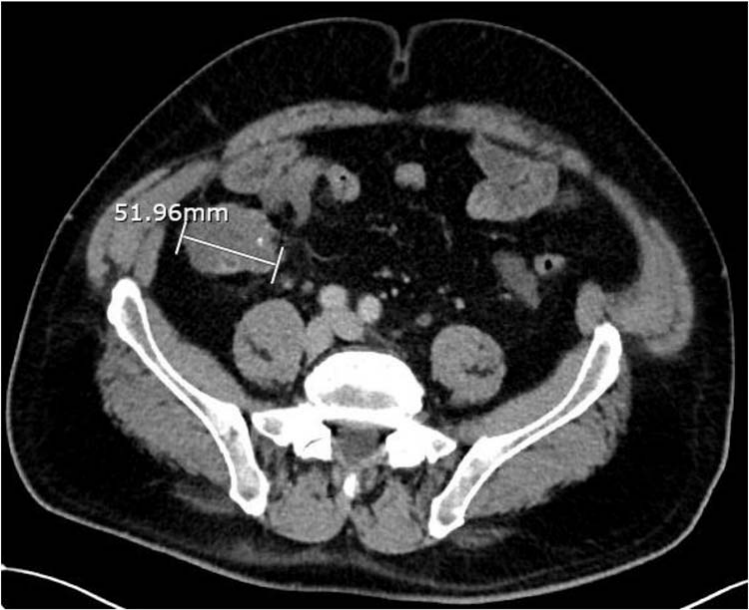
Axial CT abdomen with contrast demonstrating an appendiceal mass of maximum diameter 51.96 mm.

**Figure 2:**
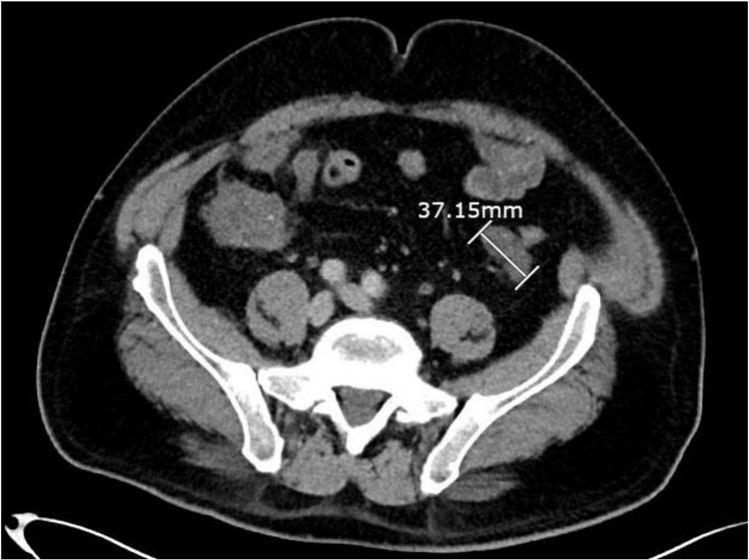
Axial CT abdomen with contrast demonstrating a peritoneal deposit posterior to the descending colon in the left iliac fossa, measuring 37.15 mm.

**Figure 3:**
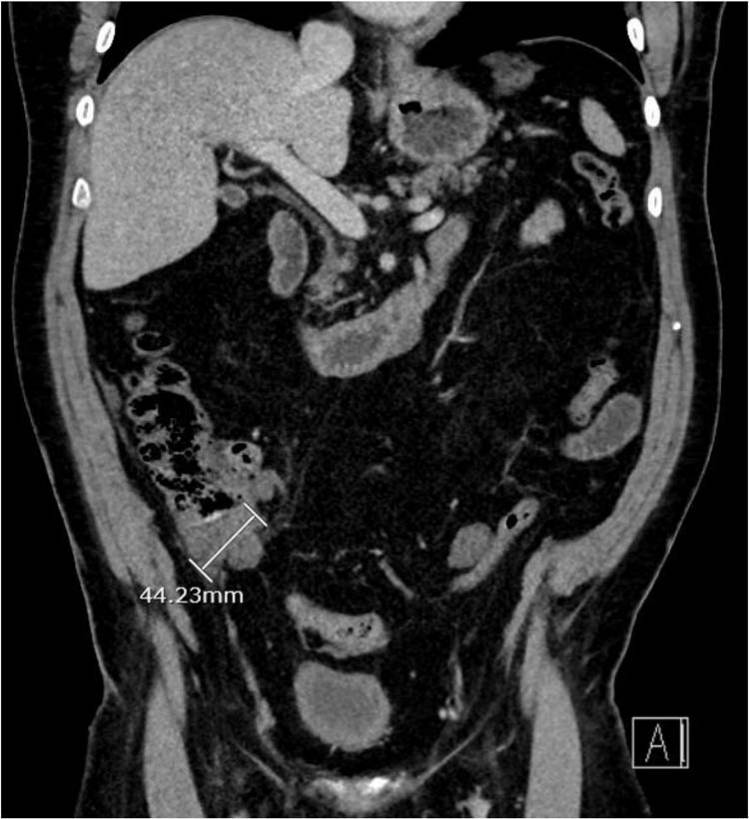
Three-month post-chemotherapy coronal CT abdomen and pelvis with contrast demonstrating a reduction in size of the primary appendiceal mass to 44.23 mm.

About 1 year later, the patient re-presented for anaemia and underwent a further CT scan. In the right iliac fossa, a 51.96 mm mass was noted such that the appendix could not be visualized separately, with a focus of calcification (Fig. 1). There was infiltration into the adjacent fat and abnormal soft tissue thickening of the peritoneal reflection along the right paracolic gutter. Multiple new peritoneal nodules in the upper abdomen were also identified (Fig. 2). The appearances were in keeping with disseminated malignancy. Following histological analysis, diagnoses of LAMN and PMP was made. The patient was initiated on mitomycin and capecitabine chemotherapy, which modestly reduced the size of the right iliac fossa mass from 51.96 mm to 44.23 mm (Fig. 3).

At 6-months post-diagnosis, the patient underwent cytoreductive surgery with right hemicolectomy and cholecystectomy, and hyperthermic intraperitoneal chemotherapy (HIPEC), having a peritoneal cancer index (PCI) of 28 and completeness of cytoreduction (CC) score of 1. Histology from specimens showed disseminated peritoneal adenomucinosis (DPAM), as well as an incidental 15 mm grade 1 well-differentiated neuroendocrine tumour (pT2, pN0, pMX), identified in the LAMN and infiltrating into the caecum. This neuroendocrine tumour was completely excised. The patient underwent a redo cytoreductive surgery with splenectomy and HIPEC 16 months later, following identification of a 10 mm recurrence in the left paracolic gutter, with a PCI of 11 and CC of 0. Histology again confirmed DPAM.

Following these operations, the patient fared well and was disease-free according to imaging. He was quoted a 5-year survival rate of over 85%.

**Figure 4:**
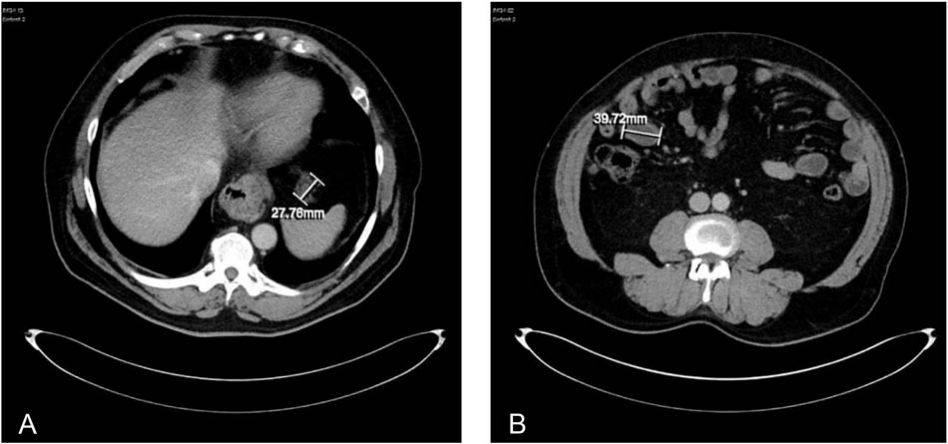
**(A)** axial CT abdomen demonstrating a serosal deposit measuring 27.76 mm. **(B)** Axial CT abdomen demonstrating a peritoneal abdominal wall deposit measuring 39.72 mm.

Unfortunately, 2 years later the patient was repeatedly admitted acutely to hospital with vomiting and physical deterioration. CT scans revealed a cystic mass surrounding the jejunal loops and re-appearances of peritoneal deposits, in keeping with aggressive progression of PMP (Fig. 4). The peritoneal multi-disciplinary team agreed not to go ahead with cytoreductive surgery and HIPEC.

It was decided to perform a laparotomy and insert a surgical gastrostomy with the aim to start total parenteral nutrition and facilitate a safe discharge home. The biopsy performed of the peritoneal deposits showed a change in morphology to grade 3 mucinous carcinoma peritonei with signet ring cells. The patient declined chemotherapy and sadly died 6 months later.

## DISCUSSION

The simultaneous presence of mucinous and neuroendocrine neoplasms in the appendix is exceptionally rare, with few reported cases [3, 4]. Collision tumours are defined as the presence of two histologically distinct tumour types adjacent to each other, but with well-demarcated margins. Our case is highly unique in that we report a 64-year-old patient who presented with acute appendicitis, and was subsequently diagnosed with PMP, LAMN and a neuroendocrine tumour within the LAMN.

PMP is a rare clinical entity, characterized by diffuse intra-abdominal mucinous implants on peritoneal surfaces. The ‘redistribution phenomenon’ describes the flow of intraperitoneal fluid and the effect of gravity on the ability for free-floating mucinous epithelial tumour cells to implant in various parts of the peritoneal cavity. The small bowel is relatively spared due to its peristaltic movements preventing tumour cells from adhering [5]. Diagnosis may be challenging, and most cases are discovered incidentally via imaging.

Due to the rarity of the disease, there is no standard for management. However, studies advocate the use of cytoreductive surgery and HIPEC for good surgical candidates [6]. A study by Youssef *et al*. showed the 5-year survival rates were 87% in patients who underwent complete cytoreduction versus 34% in those patients who underwent solely major debulking of the tumour [7]. Interestingly, as with our case, one study showed that transitions from less aggressive to more aggressive histological types are seen in association with multiple surgical interventions [8].

Our case highlights the imperative need for surveillance in patients with PMP. Even though our patient had a disease-free interval, and was quoted a favourable life-expectancy, unfortunately the disease process recurred with a change in morphological features to a more aggressive cancer type.
